# Dynamicity of brain network organization & their community architecture as characterizing features for classification of common mental disorders from whole-brain connectome

**DOI:** 10.1038/s41398-024-02929-5

**Published:** 2024-06-29

**Authors:** Nisha Chetana Sastry, Arpan Banerjee

**Affiliations:** Cognitive Brain Dynamics Laboratory, National Brain Research Centre, Gurgaon, Haryana India

**Keywords:** Biomarkers, Psychiatric disorders

## Abstract

The urgency of addressing common mental disorders (bipolar disorder, attention-deficit hyperactivity disorder (ADHD), and schizophrenia) arises from their significant societal impact. Developing strategies to support psychiatrists is crucial. Previous studies focused on the relationship between these disorders and changes in the resting-state functional connectome’s modularity, often using static functional connectivity (sFC) estimation. However, understanding the dynamic reconfiguration of resting-state brain networks with rich temporal structure is essential for comprehending neural activity and addressing mental health disorders. This study proposes an unsupervised approach combining spatial and temporal characterization of brain networks to classify common mental disorders using fMRI timeseries data from two cohorts (*N* = 408 participants). We employ the weighted stochastic block model to uncover mesoscale community architecture differences, providing insights into network organization. Our approach overcomes sFC limitations and biases in community detection algorithms by modelling the functional connectome’s temporal dynamics as a landscape, quantifying temporal stability at whole-brain and network levels. Findings reveal individuals with schizophrenia exhibit less assortative community structure and participate in multiple motif classes, indicating less specialized network organization. Patients with schizophrenia and ADHD demonstrate significantly reduced temporal stability compared to healthy controls. This study offers insights into functional connectivity (FC) patterns’ spatiotemporal organization and their alterations in common mental disorders, highlighting the potential of temporal stability as a biomarker.

## Introduction

Common mental disorders, such as bipolar disorder, attention-deficit hyperactivity disorder (ADHD), and schizophrenia, have a significant societal impact in terms of disability-adjusted life years (DALYs), years lived with disability (YLDs), and years of life lost (YLLs) [1]. Developing prevention and intervention strategies to assist behavioural psychiatrists is crucial in addressing these disorders. Neuroimaging techniques like functional magnetic resonance imaging (fMRI) and brain connectivity analytics offer promising non-invasive tools for early detection and management of these disorders [2, 3]. The spontaneous resting state dynamics of the brain, which is believed to emerge from robust metabolic and neural information processing principles [4, 5] offers a practical framework for investigating biomarkers of these disorders. However, a systematic and comprehensive approach to pattern detection and biomarker identification is lacking. This study aims to establish a unified data-driven approach to identify biologically meaningful patterns that differentiate common mental disorders and uses spatial distribution and temporal dynamics of brain networks.

Previous studies investigating alterations in resting-state brain dynamics in common mental disorders have largely focused on the strategies of functional connectome communities—the mesoscale organization of individual neural elements into motifs, circuits, or clusters [6]. These studies have reported changes in community organization such as decreased modularity of brain networks in schizophrenia and bipolar disorder [7–9]. Studies using multilayer community detection algorithms have reported higher flexibility in patients with schizophrenia [10] and ADHD [11]. A major drawback of these studies, in addition to the methodological biases of the community detection algorithms [6] is the utilization of *static* functional connectivity to detect functional connectome communities. Recent research has shown that resting-state functional brain networks undergo spontaneous, time-varying, and large-scale dynamic reconfiguration [12]. This suggests that measures assuming stationarity for the entire duration of the scan are too simplistic to capture the full extent of resting brain dynamics [13].

Dynamic functional connectivity (dFC) can exhibit non-random and non-trivial temporal structures [14] and is associated with cognitive processing, learning, attention, and performance [15–18]. The dysconnectivity observed in these disorders suggests the presence of complex spatiotemporal alterations in functional connectivity (FC) [10, 19]. This naturally raises the question: Are there alterations in dFC within the resting state functional connectome in common mental disorders and if so, can they be accurately captured and comprehensively studied? Moreover, can these patterns of dFC serve as characterization tools for common mental disorders? Stability in dFC patterns over time ensures consistent information representation in the neural connectome [20]. While temporal stability of the brain’s functional connectome has been studied extensively during resting state [21], limited research has explored alterations in the temporal stability of the whole-brain dynamic functional connectome in common mental disorders.

To estimate the temporal stability of dFC, researchers often employ methods like K-means clustering [22, 23] or Hidden Markov models [24] to identify discrete brain states and measure the switching rate or flexibility between these states. Higher flexibility suggests more frequent transitions, which leads to reduced temporal stability [25]. Graph-theoretical studies use *temporal correlation coefficient* to assess dynamic brain network stability. However, these studies are limited in effectiveness due to the ad-hoc selection of the number of states in clustering algorithms [26, 27]. An alternative perspective considers dFC as a continuous process, examining global and regional dynamics using techniques like Pearson correlation, angular distance, or dFCSpeed [21, 28, 29]. This approach captures the continuous fluctuations in dFC without relying on predefined states.

Thus, the present study has two main objectives. Firstly, it aims to examine the potential of whole brain and sub-network level measures of dFC as characterizing tools for common mental disorders. This investigation is conducted using a large cohort (*N* = 408) comprising healthy individuals as well as those diagnosed with schizophrenia, bipolar disorder, and ADHD. Secondly, the study aims to provide a comprehensive understanding of the impaired brain network mechanisms associated with these disorders by combining community-based detection and dynamics-driven characterization of the functional connectome.

## Materials and methods

### Participants and image acquisition

Overall, the study included two multicentre datasets involving 408 human participants.

### Dataset 1

fMRI data from 285 participants who participated in the University of California Los Angeles (UCLA) Consortium for Neuropsychiatric Phenomics LA5c study was downloaded (*Dataset 1*) [30, 31]. The public database was obtained via openfMRI (https://openfmri.org/dataset/ds000030/) and includes 138 healthy controls (HC), 58 individuals diagnosed with schizophrenia (SZ), 40 with ADHD and 49 with bipolar disorder (BP). Further details of the participants, image acquisition and preprocessing of the data are provided in the supplementary material.

### Dataset 2

For the replication analysis, a publicly available dataset from the centre for Biomedical Research Excellence (COBRE) was obtained [32, 33]. The neuroimaging dataset *(Dataset 2)* included resting state fMRI scans from 72 participants with schizophrenia and 74 healthy controls. Additional details regarding participant characteristics, image acquisition, and data preprocessing can be found in the supplementary material.

### Data preprocessing and parcellation

**Fig. 1:**
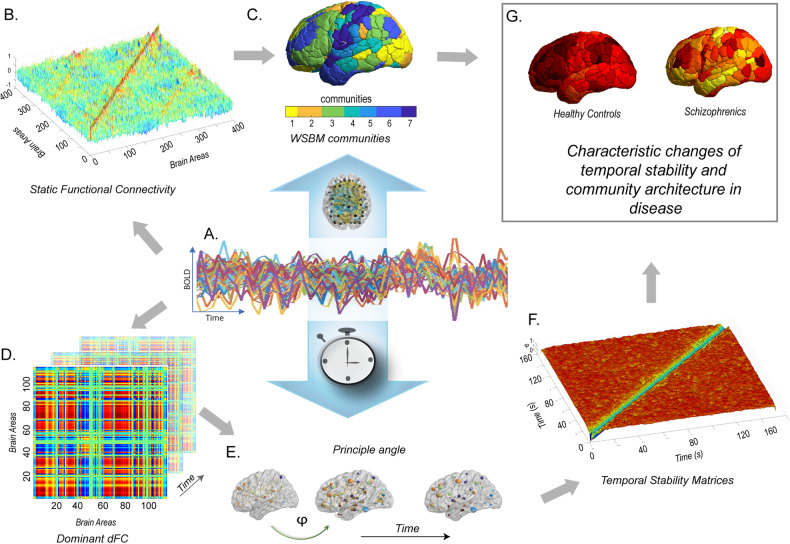
A brief overview of the methodology used in this study. **A** Shows the BOLD time series data, **B** illustrates the estimation of static functional connectivity using Pearson correlation, which is then transformed using the r-to-z method, **C** displays topographic representation of the WSBM communities. The subject-wise undirected, signed, weighted adjacency matrix serves as the input for the Weighted Stochastic Block Model (WSBM), a data-driven generative community detection algorithm that groups brain areas into *K* = 7 communities based on their stochastic equivalence. **D** Presents the matrix representation of the reduced dominant dynamic functional connectivity (dFC) patterns, denoted as D(t), computed at each time point. **E** Demonstrates the calculation of similarity between dominant dFC subspaces using angular distance or principal angle (ϕ). **F** Showcases the Time X Time temporal stability matrix, where each entry represents the principal angle $\left(\phi \left({t}_{x},{t}_{y}\right)\right)$ between dominant dFC subspaces at time points ${t}_{x}$ and ${t}_{y}$. This matrix visualizes the temporal landscape, with the principal angle ranging from 0 (indicating low angular distance) to π/2 (indicating high angular distance). Constructing a Time X Time temporal stability matrix allows us to visualize the temporal “landscape” for the entire duration of the scan. **G** We calculate the temporal stability of the dynamic functional connectome using two measures: entropy and global temporal distance. We investigate changes in community architecture in common mental health disorders such as schizophrenia, bipolar disorder, and ADHD.

The rs-fMRI images were pre-processed using the CONN toolbox (McGovern Institute for Brain Research, MIT, USA) in MATLAB (The MathWorks). The default CONN preprocessing pipeline (defaultMNI) was employed, consisting of functional realignment and unwarp, slice-time correction, outlier identification, direct segmentation and normalization, and functional smoothing. Further details are provided in the supplementary material. The final datasets used in this analysis and their group characteristics are described in Supplementary Table S1. Resting state scans of each participant were parcellated using 400 region Schaefer parcellation [34]. This atlas was chosen as it pre-allocates brain regions into resting state networks (RSNs). For each subject, mean blood oxygen level dependant (BOLD) time series (Fig. 1A) were estimated for each region over all voxels belonging to that brain region.

### Dominant dFC and temporal stability matrices

These methods have first been introduced in ref. [21] in the context of healthy ageing. For each subject, we estimate time-resolved dFC using BOLD phase coherence (Fig. 1A) [22, 35], which resulted in a matrix with size N X N X T, where *N* = 400 is the number of brain regions, T is the total number of time points (*T* = 152 for *Dataset 1*, and *T* = 150 for *Dataset 2*). First, instantaneous phases *θ (n, t)* of the BOLD signals for all the brain regions was calculated using Hilbert transform. Given the phases of BOLD signals, phase coherence between brain areas *n* and *p* at each timepoint *t*, i.e., *dFC (n, p, t)* is computed as:

1 $${dFC}\left(n,p,t\right)=\cos \left(\theta \left(n,t\right)-\theta (p,t)\right)$$

To characterize the evolution of dFC over time, we extract dominant subspace of dFC patterns (Fig. 1B), by applying principal component analysis (PCA) [21, 36].

Given a set of dominant dFC matrices (*D*_*t*_), we seek to characterize temporal stability using the similarity of dFC patterns across timepoints. We use angular distance (Fig. 1C) to estimate the similarity between dominant dFC subspaces. A detailed overview of the methodology can be found in [21].

### Measures of temporal stability

Constructing a *time X time* temporal stability matrix allows us to visualize the ‘*temporal landscape’* (Fig. 1D) for the entire duration of the scan. We introduce two distinct perspectives on measures of temporal stability—firstly, we seek to quantify temporal stability over the entire temporal landscape across all timepoints. To achieve this, we evaluate the informational content of the stability matrices by calculating entropy [21]. Entropy was used because it is a more direct measure of order and disorder in a dynamical system and provides us a measure of distinguishable temporal order that can be interpreted as the overall stability of the temporal landscape [37]. Entropy is defined by the following equation:

2 $$E=-\sum p\,{\rm{l}}{\rm{o}}{\rm{g}}(p)$$

Where *p* contains normalized histogram counts returned from “imhist.m” applied on temporal stability matrices, estimated using reduced ${D}_{{t}_{x}}$ and ${D}_{{t}_{y}}$. “imhist.m” calculates the histogram of temporal stability matrices and returns the normalized counts. Overall temporal stability is estimated as follows

3 $${TS}=1/E$$

Secondly, we seek to quantify temporal stability across successive time windows. To do this, we estimate the *global temporal distance* by taking the average of the off-diagonal elements of the temporal stability matrix. The off-diagonal elements are angular distances among dominant dFC subspaces at two successive time points, thus the term ‘*global’* signifies that the measure captures a temporally averaged snapshot of the dFC evolution.

4 $${Global\; temporal\; distance}=\frac{1}{(t-1)}\mathop{\sum }\limits_{i,j=i+1,i\ne j}^{T-1}\phi \left({t}_{i},\,{t}_{j}\right)$$

Where, $\phi$ $({t}_{i}$, ${t}_{j}$) is the angular distance entry at *i*th row and *j*th column in the temporal stability matrix, T is the total number of timepoints. *Global temporal stability (TS*_*global*_*)* is defined as the inverse of the *global temporal distance*:

5 $${{TS}}_{{global}}=\frac{1}{{Global\; temporal\; distance}}$$

### Model-based community detection using the weighted stochastic block model (WSBM) on static FC

We estimate the sFC [38] between two brain areas, *n, p* by calculating the Pearson correlation between BOLD time series of brain areas *n, p* [39] (Fig. 1E). The correlations were subsequently r-to-z transformed. The *N X N* FC matrix was represented as a network in which regions were represented by network nodes and FC between region *n* and *p* was represented by network edge between nodes *n* and *p* [40]. Thereafter, subject-wise undirected, signed, weighted adjacency matrix *(A)* was estimated for detection of community architecture. For each subject, for a given *N* x *N* adjacency matrix, we estimate WSBM and maximize the likelihood using variational Bayes algorithm described in ref. [41]. We select *k* = 7 (number of communities) [26, 42] and repeat the optimization procedure 30 times for each subject. We implement the WSBM procedure in MATLAB using freely available code and estimate a WSBM for each subject (https://aaronclauset.github.io/wsbm/) [42]. Additional details on WSBM can be found in supplementary material.

### Measures of community architecture and interaction motifs

WSBM assigns brain areas into communities (Fig. 1F). We characterize the interaction between communities using interaction motifs described in ref. [6]. One dimension on which we characterized the community interaction was the extent to which detected communities were assortative. The interaction between two communities, *r*, and *s*, can be characterized by the community densities:

6 $${\omega }_{{rr}}=\frac{1}{{N}_{r}{N}_{r}}\sum _{i\epsilon r,j\in r}{A}_{{ij}}$$

$${\omega }_{{ss}}=\frac{1}{{N}_{s}{N}_{s}}\sum _{i\epsilon s,j\in s}{A}_{{ij}}$$

$${\omega }_{{rs}}=\frac{1}{{N}_{r}{N}_{s}}\sum _{i\epsilon s,j\in r}{A}_{{ij}}$$

Where ${N}_{r}$ and ${N}_{s}$ are the number of nodes assigned to communities *r* and *s* and *A* is the adjacency matrix. Given these community densities, we classify their interactions as follows:

$${M}_{{rs}}=\left\{\begin{array}{l}\begin{array}{ll}{M}_{{assortative}},{if} \, \min \left({\omega }_{{rr}},{\omega }_{{ss}}\right) \,>\, {\omega }_{{rs}}\end{array}\\ \begin{array}{ll}{M}_{{core}-{periphery}},{if} \, {\omega }_{{rr}} \,>\, {\omega }_{{rs}} \,>\, {\omega }_{{ss}}\end{array}\\ \begin{array}{ll}{M}_{{core}-{periphery}},{if} \, {\omega }_{{ss}} \,>\, {\omega }_{{rs}} \,>\, {\omega }_{{rr}}\end{array}\\ \begin{array}{ll}{M}_{{disassortative}},{if} \, {\omega }_{{rs}} \,>\, \max \left(\,{\omega }_{{rr}},\,{\omega }_{{ss}}\right)\end{array}\end{array}\right\}$$

Although interaction motifs are defined at the level of communities, the motifs can be mapped and an analogous score for individual brain regions can be calculated. Given a region *i’s* community assignment ${z}_{i},$ its connection density to a community *r* is given by

7 $${a}_{{ir}}=\frac{1}{{n}_{r}}\sum _{j\in r}{A}_{{ij}}$$

Then the regional assortativity score is given by:

8 $${{Assr}}_{{reg}}={a}_{{{iz}}_{i}}-{\max }_{r\ne {z}_{i}}^{{a}_{{ir}}}$$

While calculating both regional and community assortativity scores, singleton communities have been excluded [6].

### Diversity index

In addition to studying interaction motif classes (assortative, core, periphery), for each motif class, we calculate how frequently it appears among community *r’*s interactions. For a *k* community partition, community *r* participates in *k – 1* interaction. The frequencies of appearance can be expressed as probabilities, ${P}_{a}$, ${P}_{c},\,{P}_{p},$ and ${P}_{d}$ (“assortative”, “core”, “periphery”) and we can then calculate the entropy as:

9 $${H}_{r}=-\left[{P}_{a}\,log\, {P}_{a}+{P}_{c}\,log\, {P}_{c}+{P}_{p}\,log\, {P}_{p}\right]$$

The entropy or Diversity Index is *0* if the community (*r*) participates in only one class and is maximized if *r* participates in all classes equally [43]. The resulting score is then assigned to all the nodes $i\in r$. We calculate this for all *k* communities and estimate mean diversity index by averaging across communities.

### Morphospace analysis

We adopt this analysis from ref. [6]. A morphospace is a hyperspace whose axes represent the features of the organism or a system. Network morphospace represents the topological properties of a network and helps visualize the richness of the topology [44]. In this study, we construct a community morphospace, whose axes are within-community ${(\omega }_{{rr}},\,{\omega }_{{ss}}$) and between-community densities (${\omega }_{{rs}}$). Each point in the morphospace represents a pair of communities, *r*, and *s*.

## Results

We report on how the community architecture and temporal characteristics of functionally connected brain networks can be used as a characterization tool for common mental disorders. The community architecture was assessed using the WSBM to uncover the mesoscale community structure from sFC (see “Materials and methods”). We used three network topology measures that indicate each brain area’s participation in assortative, core, or peripheral community interactions [43] and demonstrate their distinctness across common mental disorders. We examined temporal properties using the temporal stability of functionally relevant brain networks, employing established methods [21]. This involved capturing temporal fluctuations in dFC, constructing a *temporal landscape* with temporal stability matrices. We computed entropy and global temporal distance metrics to quantify the informational content within these matrices, serving as defining characteristics for mental health disorders such as schizophrenia, ADHD, and bipolar disorder. Section “Distribution of community architecture of brain networks across spectrum of common mental disorders” presents community architecture analysis results and section “Impaired and preserved patterns of temporal stability in common mental disorders: schizophrenia, ADHD and bipolar” focuses on temporal stability analysis for *dataset 1* [30] in both diseased and healthy cohort. To ensure validity, we replicate the pipeline with *dataset 2* [33], specifically examining participants with schizophrenia in section “Validation analysis”.

### Distribution of community architecture of brain networks across spectrum of common mental disorders

**Fig. 2:**
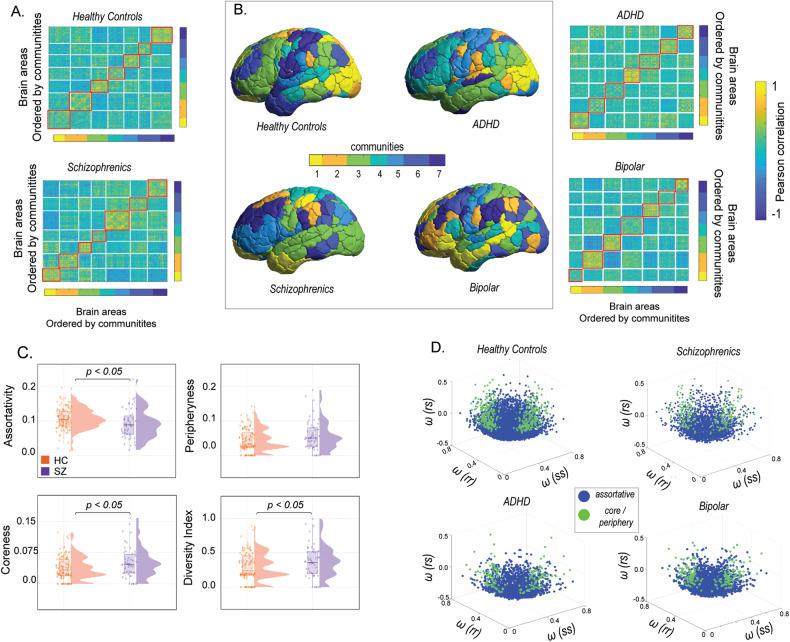
An overview of the community architecture differences between schizophrenics and healthy controls in Dataset 1. **A** Shows the static functional connectivity matrices of schizophrenics and healthy controls, with brain areas ordered by communities. **B** Presents the partition of cortical regions into 7 communities using the Weighted Stochastic Block Model (WSBM), a generative community detection algorithm that groups stochastically equivalent brain regions into communities (**C**) each pair of communities (r and s) is classified into one of three community motifs: assortative, coreness, and peripheryness. The diversity index is calculated as the average across all brain regions per subject. The violin plots indicate that in schizophrenics, communities are less assortative. **D** Illustrates the construction of a network morphospace using all pairs of communities, which are coloured according to their motif type: blue for assortative community interactions and green for core or periphery community interactions.

In *dataset 1*, a WSBM (see “Materials and methods” for details) was fitted on the adjacency matrix for each subject in the diseased and healthy cohorts. The number of communities was set at *k* = 7 based on previous studies [26, 42]. Figure 2A shows the community partitions in FC for both diseased and healthy individuals, while Fig. 2B presents a topographic representation of the communities detected with WSBM in individuals with schizophrenia, ADHD, bipolar disorder, and healthy controls. Distinct assignments of brain areas to different communities were observed in diseased and healthy controls (Fig. 2B). Community motifs reflect assortative, core, or peripheral interactions among pairs of communities. In assortative communities, the internal density of connections within subnetworks exceeds their external density whereas core-periphery organization consists of a central core which is connected to the rest of the subnetworks and peripheral nodes connect to the core but not with each other [43]. Here in the main manuscript, we report only significant group-level community interaction measures averaged across communities. Figure 2C reports significantly lower assortativity in schizophrenics (*P* = 0.0121, *t* = 2.5374) compared to healthy controls. The distributions were parametric and unpaired *t* test was used to assess significance. Rank-sum test (the distribution was non-parametric) revealed a significantly higher coreness in schizophrenics (*P* = 0.0188) compared to healthy counterparts. Additionally, the motif participation index and diversity index were computed for individual brain areas. Figure 2C reveals a significantly higher diversity index in schizophrenics (unpaired *t* test*, P* = 0.0354, *t* = *−*2.1207) indicating their communities, by and large, participate in more than one motif class. Although distinct modifications in community interaction motifs were observed for individuals with bipolar disorder and ADHD compared to healthy controls (see Supplementary S1) the results were non-significant. Next, a 3D community morphospace was constructed, where each point represents a pair of communities *{r,s}*, and the axes are defined by within-community and between-community densities, ${\omega }_{{rr}}$,${\omega }_{{rs}}$,${\omega }_{{ss}}$. Morphospace analysis revealed that individuals with schizophrenia, ADHD, and bipolar disorder favoured fewer assortative and included more core-periphery community interactions than healthy controls (Fig. 2D). Overall, the community motifs and morphospace analysis indicate community structure in common mental disorders, especially in schizophrenics is less assortative.

**Fig. 3:**
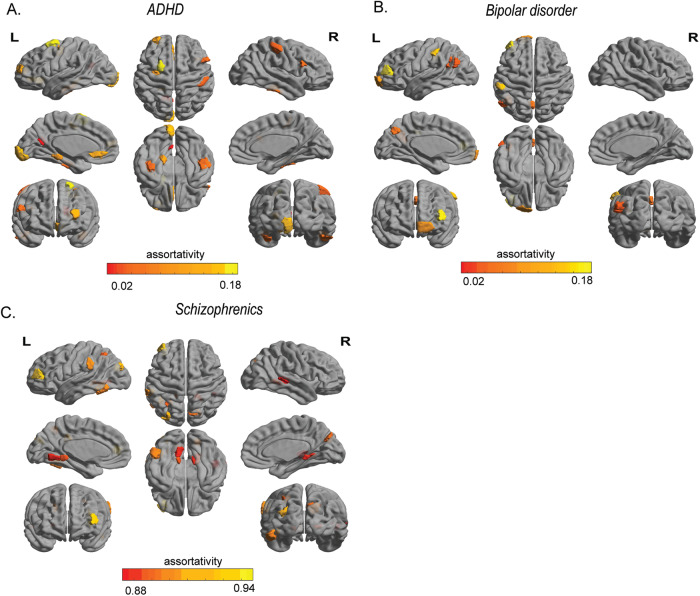
Profile of brain regions showing significant difference in assortativity between patients, age and sex-matched healthy controls. **A** ADHD, **B** bipolar disorder, **C** schizophrenia.

Next, to identify disease-specific changes in brain regions, an analogous assortativity score was calculated for each region in patients with schizophrenia, ADHD, and bipolar disorder from *dataset 1* (see “Materials and methods”) [6]. Age-matched and sex-matched healthy controls were generated for each disease group. For each brain region, a *t* test was performed between the assortativity scores of patients and matched healthy controls, with age and sex regressed out. In patients with schizophrenia, significant decreases in assortativity were observed in the peripheral visual, Dorsal attention, Ventral attention, and Temporal parietal networks (Supplementary Table S2) whereas for individuals with ADHD, significant decreases in assortativity were found in the central visual, limbic, dorsal attention, default, and control networks (Supplementary Table S4). In individuals with bipolar disorder, significant reductions in assortativity were seen in the dorsal and ventral attention, limbic, and default networks (Supplementary Table S3). Figure 3A–C shows brain-wide topography of significant assortativity scores between patients with ADHD, bipolar disorder, schizophrenia and healthy controls.

### Impaired and preserved patterns of temporal stability in common mental disorders: schizophrenia, ADHD and bipolar

To compute the temporal stability of dFC, the first step involved estimating the similarity/differences between dominant dFC subspaces by calculating the angular distance between them ($\phi \left({t}_{x},\,{t}_{y}\right)$). The resulting temporal stability matrix, spanning time X time, characterizes the collective temporal characteristics of dFC and aids in visualizing the *temporal landscape*. A low angular distance between subspaces indicates that the corresponding dFC patterns were similar in configuration, while a high angular distance suggests dissimilarity [21]. In this section, we present the results of evaluating temporal stability changes between healthy controls and cohorts with common mental disorders (such as schizophrenia, ADHD, and bipolar disorder) in *Dataset 1*.

**Fig. 4:**
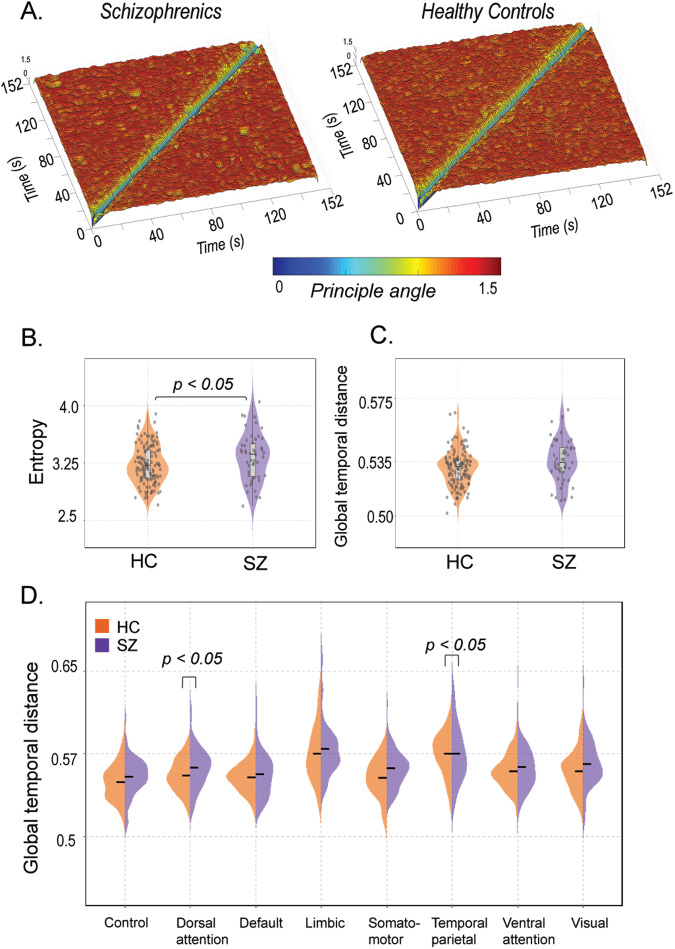
An overview of the temporal stability differences between schizophrenia and healthy controls in Dataset 1. **A** Showcases the Time X Time temporal stability matrices, visualized as the *‘Temporal landscape’*. Each entry represents the angular distance between dominant dFC subspaces at time points ${t}_{x}$ and ${t}_{y}$, for patients with schizophrenia and healthy controls. In schizophrenics, there is a global spread of shorter-lived, low angular distant (yellow hue) repeated patterns of stability (**B**) focuses on quantifying temporal stability across the entire Time X Time temporal landscape using entropy (**C**) quantifies temporal stability over successive time points using global temporal distance. Both measures indicate low temporal stability in schizophrenics. **D** Global temporal distance is estimated for all the resting state networks defined in the Schaeffer atlas, comparing schizophrenics (purple) and healthy controls (orange).

Group-level averages of temporal stability, computed on resting-state fMRI BOLD time series from healthy controls and individuals with schizophrenia, are shown in Fig. 4A. Schizophrenics exhibit shorter-lived, low angular distance (yellow hue) repeated patterns of stability, while healthy controls display a more evenly distributed pattern. Quantifying the differences, we calculated the entropy of temporal stability matrices Fig. 4B, revealing higher entropy in schizophrenics, indicating impaired temporal stability. The distributions were assessed parametrically using the Jarque–Bara test and D’Agostino–Pearson omnibus test. A two-sample *t* test revealed significant differences in entropy values between healthy controls and individuals with schizophrenia (*P* = 0.0475, *t* = −1.9963). Global temporal distance (see “Materials and methods” for details) also suggests higher values in individuals with schizophrenia. Although the results (Fig. 4C) were not statistically significant (Wilcoxon rank-sum test, *P* = 0.8902), the violin plots suggest a trend: individuals with schizophrenia exhibit higher global temporal distance compared to healthy controls. Analysing the 8 resting-state networks [34] using the same pipeline, we observed significantly higher global temporal distance (Fig. 4D) in the Dorsal attention network (*P* = 0.0153, *t* = −2.4489) (The distributions were assessed parametrically using an unpaired two-sample *t* test) and Somatomotor network (*P* = 0.0109) (The distributions were non-Gaussian; hence, the Wilcoxon rank-sum test was used), indicating significantly lower temporal stability in individuals with schizophrenia. Overall, these results indicate impaired temporal stability in individuals with schizophrenia, both at the whole-brain level and within specific networks.

**Fig. 5:**
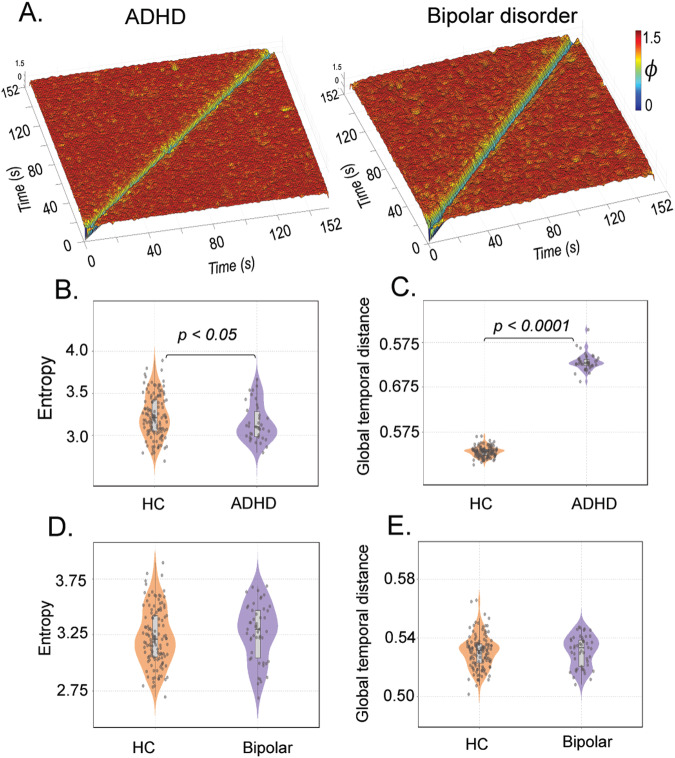
An overview of the temporal stability differences in participants with ADHD and Bipolar disorder. **A** Showcases the Time X Time temporal stability matrices, where each entry represents the angular distance between dominant dFC subspaces at time points ${t}_{x}$ and ${t}_{y}$, for participants with ADHD and Bipolar disorder. The temporal stability is quantified using two measures: entropy (**B**) and global temporal distance (**C**) in patients with ADHD and healthy controls. The results show that global temporal distance is significantly higher in participants with ADHD, indicating decreased temporal stability. Similarly, temporal stability is quantified using entropy (**D**) and global temporal distance (**E**) in participants with bipolar disorder and healthy controls.

We further examined temporal stability in patients with other common mental disorders, namely ADHD, Bipolar disorder, and healthy controls from *Dataset 1*. Whole-brain temporal stability matrices were constructed for each participant, revealing minimal spread of low angular distant (yellow hue) repeated stability patterns in both ADHD and Bipolar disorder (Fig. 5A). Participants with ADHD showed significantly lower entropy (two-sample *t* test, *P* = 0.0162, *t* = −2.4293), and higher global temporal distance (Wilcoxon rank-sum test, *P* = 2.2128e-21), indicating impaired temporal stability (Fig. 5B). In contrast, participants with bipolar disorder displayed higher entropy (two-sample *t* test, *P* = 0.3467, *t* = 0.9436) and global temporal distance (two-sample *t* test, *P* = 0.8892, *t* = 0.1395) of temporal stability matrices, although these differences were not statistically significant.

### Validation analysis

**Fig. 6:**
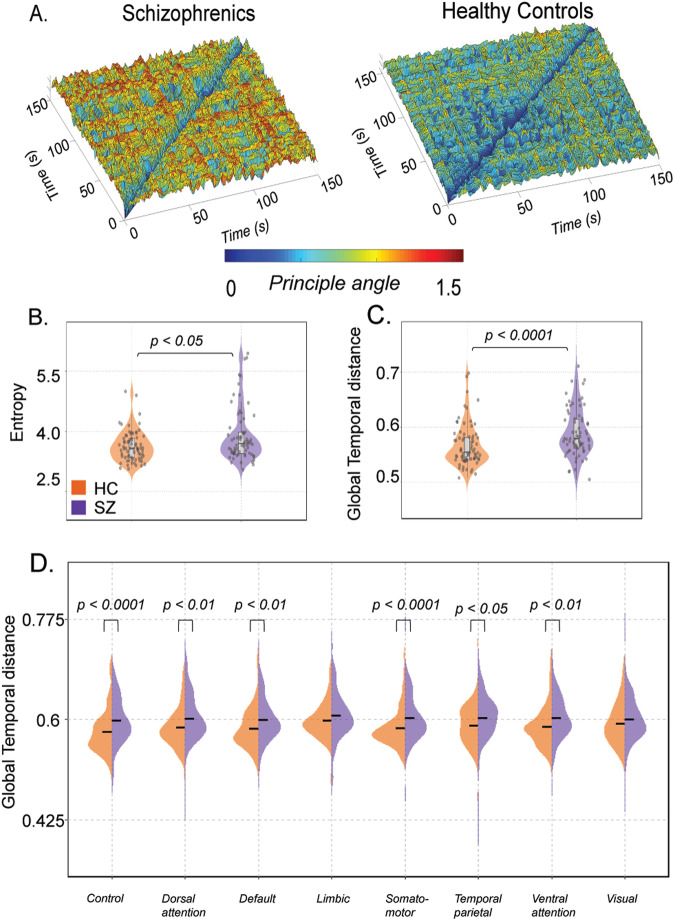
An overview of the temporal stability differences between individuals with schizophrenia and healthy controls in Dataset 2. **A** Illustrates the temporal stability matrices, visualized as a temporal landscape, for both schizophrenics and healthy controls. Each entry in the matrix represents the angular distance between dominant dFC subspaces at time points ${t}_{x}$ and ${t}_{y}$. The angular distance ranges from 0 (indicated by yellow hue) to π/2 (indicated by red hue) (**B**) quantifies the temporal stability across the entire temporal landscape using entropy (**C**) quantifies the temporal stability over successive time windows using global temporal distance. Both measures indicate a decrease in temporal stability in schizophrenics, which aligns with the findings from Dataset 1. **D** Estimates the global temporal distance for each of the resting state networks defined in the Schaeffer atlas for both individuals with schizophrenia (purple) and healthy controls (orange). The plots demonstrate a significant decrease in temporal stability in schizophrenics, particularly in the control, dorsal attention, default, somatomotor, temporal parietal, and ventral attention networks.

Furthermore, we replicated the entire analysis pipeline using *Dataset 2*. Community detection using the WSBM revealed significant differences in community interaction motifs between individuals with schizophrenia and healthy controls (see Supplementary S2A). Schizophrenia patients exhibited lower assortativity and higher coreness and peripheryness (Wilcoxon rank-sum test; *P* = 0.0483, *P* = 0.0209, *P* = 0.0043, respectively). The diversity index was also significantly higher in schizophrenia patients (*S2B*) (Wilcoxon rank-sum test, *P* = 0.0184), indicating participation in multiple motif classes. Additionally, the 3D community morphospace analysis validated these findings, specifically in patients with schizophrenia, showing fewer assortative interactions in individuals with schizophrenia (*S2C*). Temporal stability analysis confirmed impaired temporal stability in schizophrenia (Fig. 6A) patients, with higher entropy (Wilcoxon rank-sum test, *P* = 0.0373) (Fig. 6B) and global temporal distance (Wilcoxon rank-sum test; *P* = 2.5559e-04) (Fig. 6C). At the network level analysis (Fig. 6D), we observe significantly higher global temporal distance in individuals with schizophrenia in the Control network (*P* = 5.5055e-04), Dorsal attention network (*P* = 0.0057), Default network (*P* = 0.0048), Somatomotor network (*P* = 8.021e-04), Temporal-Parietal network (*P* = 0.0106), and Ventral attention network (*P* = 0.0042). The distributions were non-parametric, and significance was tested with the Wilcoxon rank-sum test. These results validate our initial findings from *dataset 1*, supporting the impaired temporal stability in schizophrenia.

## Discussion

The study aimed to explore dFC measures as tools for characterizing common mental disorders disorder. Using two datasets (*N* = 408), the WSBM [41] revealed lower assortativity and participation in multiple motifs in schizophrenia compared to healthy individuals (Fig. 2 and Supplementary S2). Comparing across diseases with matched controls, specific brain areas showed significant differences in assortativity (Fig. 3). Although altered community interactions were observed in ADHD and bipolar disorder, statistical significance was not reached (see Supplementary S1). To address this limitation, alterations in the dFC were investigated using a novel methodology [21]. This approach leverages FC dynamics to assess temporal stability in participants with ADHD, bipolar disorder, schizophrenia, and healthy controls. Entropy and global temporal distance were used as measures of temporal stability. Results showed increased entropy in the whole-brain temporal landscape and specific resting-state networks in schizophrenia, indicating decreased temporal stability of dFC (Fig. 4). ADHD participants exhibited a significant decrease in temporal stability (Fig. 5). Notably, temporal stability and community architecture alternations in schizophrenia were consistent across the two datasets used (Fig. 6).

Prior studies have shown altered community structure in mental disorders, particularly in schizophrenia. Modularity maximization studies reported decreased modularity in functional brain networks of individuals with schizophrenia [7]. Other studies using normalized mutual information found significant differences in community structure, with subcortical, auditory, and somatosensory networks being key contributors [9]. However, these studies were limited by biases associated with modularity maximization and infomap techniques [6]. To overcome these limitations, the WSBM, capable of uncovering assortative and non-assortative communities was used in this study [6]. WSBM revealed distinct communities in diseased and healthy controls (Fig. 2B and Supplementary S2A). In our analysis, we classified community interactions into assortative, core, and periphery motif classes. Results showed less assortativity, higher coreness and peripheryness in communities of individuals with mental disorders, particularly schizophrenia, compared to healthy controls (Fig. 2C, Fig. 2D and Supplementary S2). The Diversity Index indicated greater participation in multiple motif classes in schizophrenia (Fig. 2C and Supplementary S2B). Furthermore, significant changes in assortativity at the regional level were observed in schizophrenia, bipolar disorder, and ADHD. These findings provide insights into the neuropathological profiles of these common mental disorders.

Studies in schizophrenia have identified disruptions in modular structure in sensory, auditory, and visual areas [19, 45]. In our study, we found significantly lower assortativity in peripheral visual, dorsal and ventral attention, and temporal parietal networks in schizophrenia (Fig. 3C) (Supplementary Table S2). ADHD patients have shown decreased brain network integration and increased network segregation, with significant alterations in local clustering coefficients in cerebellar, frontal, motor, and temporal regions [46]. We observed significant alterations in assortativity in central visual, limbic, default, and somatomotor networks in ADHD patients (Fig. 6A) (Supplementary Table S4). Studies on bipolar disorder have identified alterations in brain network topology in frontoparietal and limbic networks [47]. Similarly, our findings indicate significantly lower assortativity in default and dorsal attention networks, as well as limbic networks, in bipolar disorder patients (Fig. 3B) (Supplementary Table S3). Overall, these region-level alterations align with previous studies and suggest alterations in community structure play a role in the neuropathology of common mental disorders. Although our attempts to replicate community detection and interaction motif analysis in ADHD and bipolar disorder patients did not yield statistically significant results, we attribute this to the limited number of participants and the dependence of most community detection algorithms on sFC. It is crucial to acknowledge the limitations of sFC in comprehensively capturing the dynamic nature of brain activity [13]. Therefore, we focused on estimating the temporal stability of the resting-state dynamic functional connectome to explore its dynamic characteristics and alterations in common mental disorders. Our method, developed from ref. [21] captures dFC patterns without predefined nodes or edges, ensuring high temporal resolution and accurate temporal stability estimation over traditional graph-theoretical approaches. The method involved extracting dominant dFC patterns at each time point, projecting them into dFC subspaces using PCA [48], and assessing the similarity of dFCs through angular distance (Fig. 1C). We introduce two measures to quantify temporal stability: (1) Entropy, which estimates temporal stability across the entire temporal landscape and all time points, and (2) Global temporal distance, which measures temporal stability across successive time points. While previous studies have explored regional variations in temporal stability [49] our study extends this exploration by quantifying temporal stability at both the whole-brain and brain network level. Our key finding is a significant decrease in temporal stability among individuals with schizophrenia at both the whole-brain and network levels. In both *Dataset 1* and *Dataset 2*, we observed increased entropy (Figs. 4B and 6B) and global temporal distance (Fig. 4C) (Fig. 6C) of the whole-brain functional connectome in individuals with schizophrenia, indicating impaired temporal stability as a marker of the disease. Furthermore, several sub-networks, including the dorsal attention, somatomotor, limbic, ventral attention, control, and temporal-parietal networks, exhibited impaired temporal stability (Figs. 4D and 6D). One earlier study [28] found that patients with schizophrenia exhibited significantly increased temporal variability (decreased temporal stability) of dFCs in subcortical regions, such as the thalamus, palladium, and visual areas during resting state. Similarly, using flexibility measures, few groups [10, 49, 50] have observed significant impairment in temporal stabilities of dFC in multiple brain areas, including the thalamus, visual areas among individuals with schizophrenia. In line with these previous studies, our results emphasizing the significant impairment in temporal stability of dFCs at both the whole-brain and network level have contributed towards a comprehensive understanding of the temporal stability differences observed in schizophrenia. ADHD is characterized by dynamic reconfiguration of the functional connectome, with increased temporal variability in the default-mode network and lower temporal variability in subcortical regions reported in previous studies [28, 51, 52]. Our findings show a significant increase in the global temporal distance of the whole-brain functional connectome in ADHD patients, indicating decreased temporal stability (Fig. 5B). Similarly, there are multiple studies reporting shared similarities in temporal stability alterations between bipolar disorder and schizophrenia [25, 53, 54]. Although our results show changes in temporal stability in individuals with bipolar disorder, the results were not statistically significant. Overall, our findings, validated across two datasets, highlight a significant widespread decrease in the temporal stability of dFC in common mental disorders, especially in schizophrenia. These alterations in dynamic functional network configurations, captured by temporal stability measures, provide a comprehensive representation of brain dynamics and reflect the dynamic nature of mental disorders. They hold promise as potential biomarkers for common mental health pathologies, particularly in ADHD and schizophrenia, where mental state fluctuations are more dynamic compared to bipolar disorder. One limitation of our study pertains to the unequal distribution of samples between diseased and healthy cohorts within *Dataset 1*, thereby restricting the interpretability of our findings but certainly improvable by future studies. Another methodological limitation of our study is with WSBM. WSBM requires the user to specify the number of communities (K). Based on previous studies, we chose *K* = 7, but this choice may impact the results [26, 42].

In conclusion, our study emphasizes the importance of dFC and temporal stability in characterizing common mental disorders. While WSBM reveals distinct patterns in the community architecture of functionally connected brain networks between diseased and healthy controls, it faces challenges in differentiating between specific disorders. However, temporal stability analysis using angular distance, global temporal distance, and entropy calculations uncovers impaired stability in schizophrenia and ADHD, while bipolar disorder exhibits non-significant differences. These findings highlight the important role of dFC and temporal stability in characterizing common mental disorders, providing insights into underlying mechanisms and potential diagnostic markers.

### Supplementary information

Supplementary material

## Data Availability

The datasets generated during the analysis pipelines in the present study are available from the corresponding author on reasonable request. The codes for ANOthe analysis carried out in this paper are available on https://bitbucket.org/cbdl/workspace/projects/DFC. We implement the WSBM procedure in MATLAB using freely available code and estimate a WSBM for each subject (https://aaronclauset.github.io/wsbm/).
